# Assessing Fall Risk, Prevention Knowledge, Behavior and Social Support Among Older Adults: Insights from a Hospital-Based Study in Riyadh

**DOI:** 10.3390/healthcare14081109

**Published:** 2026-04-21

**Authors:** Anwar Alhashem, Norah Aldarwish, Rahaf Almoqbel, Reem Alsaeed, Sabba Alanazi, Mohammed S. Khusheim

**Affiliations:** 1 Department of Health Sciences, College of Health and Rehabilitation Sciences, Princess Nourah bint Abdulrahman University, P.O. Box 84428, Riyadh 11671, Saudi Arabia; amalhashem@pnu.edu.sa (A.A.); nourah.aldarweesh@gmail.com (N.A.); rahafsts@gmail.com (R.A.); 2 Department of Nursing, Prince Mohammed Bin Abdulaziz Hospital, Riyadh 14214, Saudi Arabia; saba401789@gmail.com; 3 College of Medicine, Alfaisal University, P.O. Box 50927, Riyadh 11533, Saudi Arabia; khusheim.mohammed@gmail.com; 4 Seha Virtual Hospital, Ministry of Health, Riyadh 12382, Saudi Arabia

**Keywords:** falls, older adult, fall risk, fall prevention, STEADI, Saudi Arabia

## Abstract

Background/Objectives: Falls are a significant public health issue and the second most common cause of injury and death worldwide. The risk is exceptionally high for older adults, and fall-related injuries can significantly affect their well-being and lead to pain, disability, and loss of independence, necessitating urgent redressal of this issue. This study assesses the fall risk among older patients at a referral hospital in Riyadh, Saudi Arabia. Methods: This cross-sectional study included 246 older adults aged 65 years and older from the Internal Medicine Department. Data were gathered through a survey addressing sociodemographic characteristics, fall risk assessment, prevention knowledge, behaviors, and social support. Results: A high fall risk and good knowledge of fall prevention were observed in older adults. A weak-to-moderate positive correlation was found between behavior and fall risk. Correlations were observed among age, fall prevention behavior, educational level, and fall prevention knowledge. Conclusions: While a weak-to-moderate positive association was found between fall risk and positive behaviors, no statistically significant association was observed between fall risk and fall prevention knowledge or social support. This indicates that factors other than knowledge and social support may play a critical role in influencing fall risk. Behavioral interventions alone may not reduce fall incidence sufficiently unless other underlying factors are addressed.

## 1. Introduction

The World Health Organization defines a fall as an accidental drop to a lower surface. Falls are a major concern worldwide, leading to severe injuries and potentially fatal outcomes, particularly among older adults. Aging is a major risk factor of falls. As the population ages, the frequency of falls also increases [1].

The fall rates among older adults are higher in Saudi Arabia than in other countries. A study reported a prevalence of 57.7% in Saudi Arabia, which is more than double that reported in Brazil (25.8%). This study also showed that falls are more frequent in Saudi Arabia than in Japan, India, or Qatar, with a prevalence of approximately 34% [2]. Despite the prevalence of falls among older adults, there is an apparent gap in the literature on falls among older adults in the Middle East [3].

One initiative suggested by the Centers for Disease Control and Prevention to help prevent falls was the Stopping Elderly Accidents Deaths and Injuries (STEADI) initiative, which was developed in collaboration with American and British geriatric societies.

The STEADI initiative is meant to guide healthcare practitioners in helping older adults with a history or risk of falling. It includes three main components: (1) screening for fall risk; (2) assessment of modifiable risk factors; and (3) intervention using clinical and community approaches [4]. When these aspects are combined, they can have a significant influence on fall prevention, enhancing health outcomes, and lowering healthcare costs [4].

Recognizing the need for further research on fall prevention strategies, specifically for the older adult population in Saudi Arabia, this study aimed to bridge this gap by assessing fall risk among older adult patients at a referral hospital in Riyadh, Saudi Arabia, using the STEADI program and social support assessment tools.

## 2. Materials and Methods

### 2.1. Study Design and Settings

Conducted between December 2023 and May 2024, this study employed a cross-sectional descriptive design. Data collection began once IRB approval was obtained and lasted for up to one month.

### 2.2. Population and Sampling

This study included older adult patients recruited from the internal medicine department of a referral hospital in Riyadh. The participants were provided with a written consent form, and the interviews were conducted once signed. The interviews were not recorded; instead, the responses were marked on the questionnaire.

The study used convenience sampling, a nonprobability sampling method. Participants were recruited based on their availability in the clinic at the time of data collection and were then asked to participate.

The sample consisted of all opened files in the internal medicine departments of any older adult aged 65 years and older. The required sample size was calculated using the following formula: $n=\frac{Z^{2}P\left(1-P\right)}{d^{2}}$. A confidence level of 95% was assumed (Z) to be 1.96, a margin of error (d) of 0.05 was used, and in the absence of precise local estimates, a conservative proportion of 0.2 was assumed. This yielded the required sample size of 245.86, rounded to 246 participants. The calculation was performed and verified using the n4Studies statistical tool (n4Studies Co., Bangkok, Thailand) developed by Ngamjarus and Pattanittum [5].

### 2.3. Inclusion and Exclusion Criteria

The study surveyed Saudi citizens aged 65+ years with cognitive capacity, who are patients of internal medicine at the hospital, and their Arabic primary caregivers aged 18+ years, excluding those who were not primary caregivers or non-Arabic speakers.

### 2.4. Study Instrument

To gather primary data, we conducted 15 min in-person interviews and a survey covering demographic characteristics, fall risk assessment, fall prevention behaviors and knowledge, and social support.

The sociodemographic section covered age, gender, and education level. The fall risk assessment section, based on the STEADI’s “Stay Independent” Brochure [6], comprised 12 questions. The fall prevention knowledge section included eight questions from STEADI’s “Check For Safety” Brochure [7] and the CDC’s “What You Can Do To Prevent Falls” Brochure [8], with one question addressing the Wudu area, a wet ritual for Muslims to purify themselves before prayer [9]. The instrument featured 12 questions on fall prevention behavior: ten from the Falls Behavioral Scale for the Older Person [10], and two concerning shoes and exercise. In addition, the instrument contained 7 questions to assess the social support from the online support group scale [11]. Supplementary Table S1 contains the complete study instrument, including item wording, response options, scoring, and the identification of reverse-coded items.

### 2.5. Reliability and Validity

The tool was translated into Arabic using a forward–backward translation technique, and the Arabic version was used during the interviews. Furthermore, validity was assessed for each question in the tool, and it demonstrated an r-value of 0.3 or higher and a *p*-value of less than 0.05, indicating validity.

The reliability of the study scales was assessed using Cronbach’s alpha test. For the fall risk scale, one item (I don’t feel the need to hold onto furniture when walking at home) was removed, resulting in a Cronbach’s α of 0.58. Similarly, one item was removed from the fall prevention knowledge scale (Do well-fitted shoes prevent falling?), yielding an α of 0.57. The behavior scale initially demonstrated low consistency; therefore, three underperforming items (I hurry when I do things, I don’t notice if there’s a spill on the floor, I don’t worry about walking carefully outside) were removed. Following these deletions, the Cronbach’s α improved to 0.60. The social support scale demonstrated good internal consistency (α = 0.88) without the need to remove any items. Detailed internal consistency analysis of the study scales presented in Supplementary Table S2.

### 2.6. Data Analysis

The study utilized JMP, 17th Edition (SAS Institute Inc., Cary, NC, USA) [12] and Microsoft Excel (Version 2503) for data cleaning and analysis. Descriptive statistics were used for numerical and categorical variables and pie and bar graphs were used to summarize the data. Inferential statistics, including correlation, linear regression, and chi-square tests were used to draw inferences about the older adult population. Monte Carlo *p*-value, Cramer’s V, and *p*-values of less than 0.05 were reported.

Sociodemographic characteristics of age were collected as numerical variables; however, for data analysis, another variable was calculated for age in categories that were youngest-old (aged 65–74 years), middle-old (aged 75–84 years), and oldest-old (aged 85 years and above), selected in alignment with Lee et al. [13] study targeting a similar age group.

A scoring system was used to categorize the responses to the questionnaire. For the fall assessment section (12 questions), the total score was determined by adding up the “Yes” answers, which yields a maximum of 14 points. For this study, Questions (3), (4), and (6) were reversed, resulting in “No” answers.

The knowledge section included 8 questions with a maximum score of 14, with the answer “Yes” in Questions 2–5 and 7 and 8 receiving 2 points each. The given scores were based on their significance and repetition in the STEADI’s Safety Brochure [7] and What You Can Do to Prevent Falls Brochure [8].

The fall prevention behavior section had 12 questions with four possible answers for each, with the minimum and maximum possible scores being 12 and 48, respectively. Categories were created by dividing the difference between the minimum and maximum scores (36 points) by 3 for each category.

The social support section used an Online Social Support Scale [11]. Out of the 41 questions divided into four sections, Questions 1, 3, and 6 were chosen from the esteem/emotional section, Question 14 from the social companionship section; and Questions 21, 22, and 24 from the informational section [11]. Each question was scored out of 5, with a cutoff point based on quartiles: (0–25%) denotes poor, (26–75%) denotes fair, and (76–100%) denotes high.

### 2.7. Ethical Consideration

IRB approval was obtained from Prince Mohammad bin Abdulaziz Hospital (PMAH) under log number 24-008. The study required participants to complete a written consent form and only those who completed the study were recruited. The participants were informed that their participation was voluntary, that they could withdraw at any time, and that their personal information would remain confidential.

## 3. Results

Table 1 summarizes the sociodemographic characteristics of the older adults. The majority (82.52%) of participants were between 65 and 74 years of age, with males accounting for the highest percentage (52.85%).

Table 2 reports the total scores for fall risk, knowledge, behavior, and social support. The highest mean score was observed in the domain of social support (27.1 ± 5.79), whereas the lowest mean score was recorded in the fall risk domain (5.3 ± 2.82). Notably, both fall risk and knowledge domains had possible scores ranging from 0 (minimum) to 12 and 13 (maximum), respectively. At the hospital, a predominant proportion (85%) of older adults were at risk of falls, while the remaining 15% demonstrated resilience to such risks.

**Figure 1:**
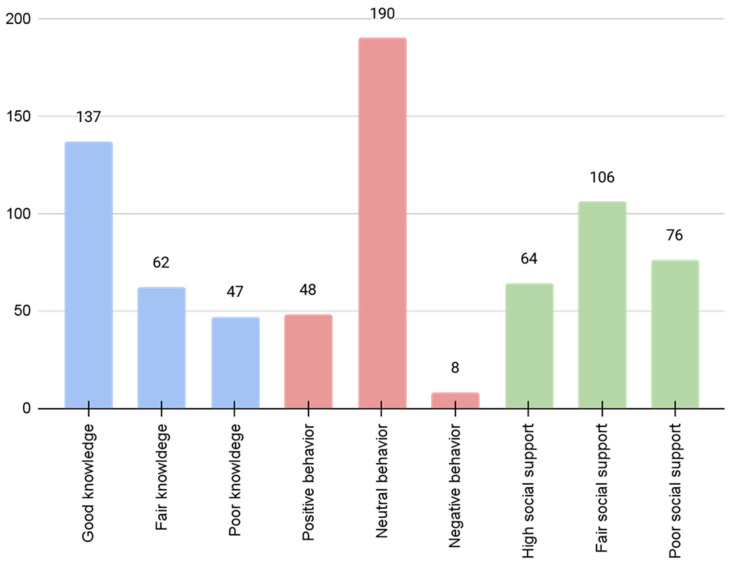
Fall prevention knowledge, behavior, and social support among older adults (N = 230). Blue bars indicate knowledge levels (good, fair, poor), red bars indicate behavior (positive, neutral, negative), and green bars indicate social support (high, fair, poor).

Figure 1 depicts fall prevention knowledge, behaviors, and social support. The analysis revealed that a significant proportion (n = 137) of participants possessed a good level of knowledge regarding fall prevention strategies. However, the behavioral component exhibited by the majority (n = 190) demonstrated neutral or moderate levels of adherence to preventive practices.

Table 3 displays the participants’ responses to the fall risk assessment. Most participants (62%) had not experienced a fall in the past year, 61% were not advised to use a cane, and only 19% reported taking medicines to aid sleep or improve mood.

Table 4 shows the participants’ responses to the knowledge section. Most of the participants recognized the link between a lack of exercise and weakness (61.79%), proper lighting (80.49%) and footwear (79.67%), and the use of grab bars (78.05%) and slip mats (81.71%) to mitigate fall risk.

Table 5 shows the participants’ responses to the behavioral section. The results show that 42.28% of the participants had never discussed fall prevention strategies. Nearly half of the participants always adjusted lighting (49.19%) and checked shoe soles before purchasing them (51.22%), and only 10.57% reported never wearing non-slip shoes at home.

Table 6 shows the participants’ responses to the social support section. Most participants expressed feeling cared for (35.77%) and encouraged (30.89%), whereas a significant number of participants reported receiving positive comments (32.52%) and belonging to supportive groups (32.93%) “a lot.” Additionally, nearly four out of ten participants (36.99%) answered “pretty often” indicating that others readily offered advice when needed.

Linear correlation (r) analysis was conducted (Table 7) to examine the relationship between fall risk and knowledge, behavior, and social support regarding fall prevention. The results revealed a weak-to-moderate positive correlation between fall risk and behavior (r = 0.35, *p* < 0.05), indicating a statistically significant but relatively modest association. Furthermore, there were no significant correlations between fall risk and knowledge (r = 0.0037, *p* = 0.9539) or social support (r = 0.11, *p* = 0.0647).

Table 8 displays a simple linear regression model for predicting fall risk based on behavior regarding fall risk and behavior (F = 35.5468, *p* < 0.0001, R square = 0.127159). Regression analysis showed a statistically significant association between behavior and fall risk; however, the proportion of explained variance was relatively low, indicating limited predictive power.

Table 9 presents the associations between age, prevention knowledge, behavior, and social support. Age group data are presented as the observed n (row %), with the expected cell frequency shown below each value. The majority of participants (82.52%) were aged 65–74 years and 57.14% had good knowledge. Age was significantly associated with fall prevention behaviors (Mcp = 0.0087, Cramer’s V = 0.184).

Table 10 illustrates the links between sex, fall prevention knowledge, behavior, and social support. The female and male participants had similar levels of knowledge (55.17% and 56.15%, respectively). Both sexes had fair social support, with no statistically significant association between gender and fall prevention knowledge, behavior, and social support (*p* > 0.05).

Table 11 shows the association between educational level and prevention knowledge, behavior, and social support. Undergraduate participants exhibited the highest level of good knowledge (67.27%) and lowest level of poor knowledge (12.73%). The behavioral scores for the undergraduate and graduate groups were similar, with most participants exhibiting fair behavior (81.82% and 82.14%, respectively). Graduate participants reported the lowest levels of social support (17.86%). Only educational level and fall prevention knowledge were statistically significant (*p* < 0.05).

## 4. Discussion

### 4.1. Assessing the Proportion of Older Adult Patients Who Are at Risk of Falling

The STEADI initiative utilizes a two-step approach to identify older adults at risk of falls [4]. The first method involves a brief screening tool comprising three core questions; an affirmative response to any question automatically classifies the patient as high-risk [4]. The second method leverages the Stay Independent Brochure, a self-administered assessment tool with 12 items; this instrument not only identifies fall risk (indicated by a score ≥ 4) but also provides valuable insights into specific risk factors and potential clinical concerns [6]. Responses to individual questions within the brochure can inform patient care pathways by guiding referrals and treatment recommendations such as physical therapy or ophthalmology consultations [14]. The initial screening phase serves as a crucial gateway for the efficient identification of individuals requiring further comprehensive assessments and targeted interventions [14].

The study identified a considerable proportion of older adult patients as being at risk of falls, with a mean score exceeding the cut-off point given in the Stay Independent Brochure. This finding aligns with prior research by Moran et al. [15] in which a similar mean fall risk score was observed with the same instrument administered by nurses, indicating consistency in fall risk identification using the Stay Independent Brochure.

### 4.2. Assessing Fall Prevention Knowledge, Behavior, and Social Support of Older Adults

This study considers the cultural aspects of older adults’ lives by asking them about their knowledge of specific prayers and Wudu (washing up before prayer). Knowledge of preventive measures among older adults is relatively high. One study conducted in Brazil used the Falls Risk Awareness Questionnaire [16], with the mean falling near the middle and leaning towards the positive side.

Health education plays an important role in improving knowledge, raising awareness, and modifying behavior. It may even be a crucial factor in fall prevention behaviors. In particular, when integrated with efforts to promote healthy behaviors and environments, and manage comorbidities, it aligns with the WHO recommendations for promoting healthy aging [16]. Programs such as the STEADI integrate educational and clinical interventions to prevent falls among older adults, including referrals to physical therapists or ophthalmologists, following knowledge and behavioral assessments in the first step [4].

As previously mentioned, modifying behaviors may be considered a protective factor against falls in older adults [17]. In the current study, the behavior section focused on what older adults currently do regarding preventative behaviors, such as holding onto handrails or adjusting lights in the household, as well as risky behaviors, such as moving too fast or not noticing spills on the floor. These behaviors, in addition to others such as inattentiveness, constant imbalance, and quick movements, give older adults little time to plan their movements patiently, and could accidentally lead to falls [18].

The results showed that the preventive behaviors of older adults exhibited a balanced spread around the middle, with the mean being close to the median. This study focused solely on outpatients, whereas a previous study [18] compared residents of nursing homes to community dwellers. They found that nursing homes residents exhibited more preventative behaviors than community dwellers. This could be attributed to nursing homes being well-established facilities prepared by healthcare professionals [18], which may indicate a deficiency in knowledge or support for community dwellers.

A supportive environment is an important factor that could influence behavior and knowledge and ultimately reduce fall risk among older adults. As mentioned previously, living in a prepared healthcare facility increases the likelihood of adopting preventive behaviors [18]. Healthy and successful aging is brought about by active engagement in life through social support, participation in various activities, and the ability to maintain physical function [19]. Social support networks function as protective factors that mitigate the negative health consequences associated with stressful life events such as falls [19].

The participants in the current study had average social support scores within the intermediate range. Because the participants were all outpatients in the internal department, this suggests that they were more independent in their daily lives. The need for a caregiver or large social support network may indicate a higher fall risk [16]. Araújo et al. [16] investigated older adults’ perceptions and knowledge. They found that those with higher social support perceived fall risk as less of a threat and had less knowledge of fall prevention than those who lacked social support. This could be attributed to the perception of safety provided by their support network, potentially leading to increased fall risk behaviors [16].

### 4.3. Association Between Level of Participants’ Fall Risk and Fall Prevention Knowledge, Behaviors, and Social Support

This study did not find any correlation between fall risk and the level of preventive knowledge among older adults, suggesting that knowledge alone may be insufficient to prevent falls. Similarly, ref. [20] argued that prevention knowledge is insufficient; healthcare practitioners must address attitudes such as fear of losing independence, emphasize the importance of fall prevention, and focus on improving older adults’ attitudes while providing practical fall prevention services to effectively influence fall prevention behavior. Furthermore, in a controlled randomized trial, Taylor et al. [21] found that personalized education tailored to the needs of older adults was significantly more effective than generalized knowledge provided to them. This finding suggests that personalized fall prevention knowledge improves adherence to fall prevention behaviors [21].

Although this study found a statistically significant correlation between fall risk and behavior, the magnitude of this association was relatively small, indicating that behavior alone may not be a strong predictor of fall risk among older adults. Moreover, the explained variance was relatively low, indicating that behavior accounted for only a low proportion of the fall risk (almost 13%). This suggests that a substantial proportion of fall risk may be influenced by other factors not included in this model, such as health status, medication use, comorbidities, and household hazards. This finding may indicate that older adults with a higher perception of their risk of falling are more likely to adopt cautious and preventive behaviors. Similar findings were reported by Clemson et al. (2003) who clarified that older adults who had fallen before or were at risk of falling made safer behavioral choices than those who had not reported a fall [22].

In our study, while older adults reported receiving emotional, informational, and social encouragement, this did not directly affect fall risk. This contrasts with the findings of Xiao et al. [23] in their cross-sectional study involving community-dwelling older adults with functional limitations. They found that greater social support was independently associated with a lower fall risk, even after adjusting for other variables, such as age and living circumstances [23]. This contrast may be due to differences in population characteristics or measurement methods. Further research is required to clarify the role of social support and its influence on the risk of falls among older adults.

### 4.4. Association Between Participants’ Sociodemographics and Fall Prevention Knowledge, Behaviors, and Social Support

Most participants were between 65 and 74 years of age and more than half had good knowledge of the subject. This could be because younger older adults are more educated and willing to practice these behaviors.

Good knowledge was distributed equally between both genders, in addition to fair social support and practicing neutral behaviors.

Undergraduate participants displayed the highest levels of good knowledge and the lowest levels of poor knowledge. The behavioral scores for both the undergraduate and graduate groups were similar, with most participants showing fair behavior. Graduate participants reported the lowest level of social support.

### 4.5. Study Limitations

This study has several limitations. The use of convenience sampling may have introduced a selection bias, as participants were recruited based on availability rather than random selection. Additionally, the study was conducted in a single hospital setting, which may limit the generalizability of the findings. Therefore, the results should be interpreted with caution and may be more applicable to older adults in similar hospital-based settings. Furthermore, the data collection instrument did not include a direct assessment of key variables, such as comorbidities, medication use, mobility limitations, frailty, and visual impairment, which are well-established predictors of fall risk. The absence of these variables limited our ability to account for potential confounding effects. Lastly, the internal consistency of the fall risk and knowledge scales was relatively low, indicating a potential measurement error. This may be partly attributable to the multidimensional nature of these scales, as the items capture diverse aspects rather than a single underlying construct.

## 5. Conclusions

The present study indicates that a significant proportion of older adults in Riyadh are at an elevated risk of falls despite possessing good knowledge regarding prevention strategies. The association between behavior and reduced fall risk was weak to moderate, whereas knowledge and social support did not demonstrate a significant correlation. This underscores the necessity of investigating the determinants of falls beyond knowledge and social support, and the importance of targeted interventions, especially for older or less educated individuals.

Given the multifactorial nature of fall risk, future research should incorporate critical clinical factors such as comorbidities, medication usage, mobility restrictions, frailty, and visual impairments.

Health educators should extend their focus beyond disseminating information and exploring the additional factors that may contribute to falls. They should participate in further research to identify these factors and develop comprehensive prevention strategies. At the policy level, the integration of fall prevention into routine health assessments should be advocated to mitigate fall-related harm in the target population.

## Figures and Tables

**Table 1:** Sociodemographic characteristics of older adults.

Characteristics	N	% of Total
Age		
65 ≤ 74	203	82.52%
75 ≤ 84	34	13.82%
≥85	9	3.66%
Gender		
Female	116	47.15%
Male	130	52.85%
Highest level of education		
No formal education	72	29.27%
Diploma or school education	91	36.99%
Undergraduate degree (Bachelor’s degree)	55	22.36%
Graduate degree (Master’s or doctorate degree)	28	11.38%

**Table 2:** Total scores of fall risk, knowledge, behavior, and social support.

Total Scores	Mean	Std Dev	Min	Max	Median
Fall risk	5.3	2.82	0	12	5
Knowledge	9.1	2.98	0	13	10
Behavior	22.7	4.19	9	32	23
Social support	27.1	5.79	7	35	27

**Table 3:** Distribution of responses to fall risk section.

Question	Yes		No	
	N	%	N	%
I have fallen at least once in the past year.	93	37.80%	153	62.20%
I use or have been advised to use a cane or walker to get around safely.	95	38.62%	151	61.38%
I feel very steady when I’m walking.	139	56.50%	107	43.50%
I am worried about falling.	117	47.56%	129	52.44%
I don’t push with my hands to get up from a chair.	79	32.11%	167	67.89%
I have some trouble stepping up onto a curb.	136	55.28%	110	44.72%
I often have to rush to the toilet.	101	41.06%	145	58.94%
I have lost some feeling in my feet.	82	33.33%	164	66.67%
I take medicine that sometimes makes me feel light-headed or more tired than usual.	96	39.02%	150	60.98%
I take medicine to help me sleep or improve my mood.	46	18.70%	200	81.30%
I often feel sad or depressed.	81	32.93%	165	67.07%

**Table 4:** Distribution of responses to fall prevention knowledge section.

Question	Yes		No		I Don’t Know	
	N	%	N	%	N	%
Is it possible to talk to your doctor about your fear of falling or if you feel unsteady?	102	41.46%	88	35.77%	56	22.76%
Can the lack of exercise lead to weakness and increase the risk of falling?	152	61.79%	54	21.95%	40	16.26%
Do regular eye exams reduce fall risk?	130	52.85%	55	22.36%	61	24.80%
Do lighting and handrails on staircases affect fall prevention?	198	80.49%	33	13.41%	15	6.10%
You can prevent falling in the kitchen by keeping things used often on lower shelves.	99	40.24%	80	32.52%	67	27.23%
You can prevent falling in the bathroom by putting grab bars next to the bathtub and toilet.	192	78.05%	41	16.67%	13	5.28%
You can prevent falling during wudu by putting non-slip mats near wudu area.	201	81.71%	24	9.76%	21	8.54%

**Table 5:** Distribution of responses to fall prevention behavior section.

Question	All the Time		Most Days		Some of the Days		Never	
	N	%	N	%	N	%	N	%
When I stand up, I pause to get my balance.	72	29.27%	75	30.49%	60	24.39%	39	15.85%
I talk with someone I know about things I do that might help prevent a fall.	30	12.20%	40	16.26%	72	29.27%	104	42.28%
I adjust the lighting at home to suit my eyesight.	121	49.19%	68	27.64%	38	15.45%	19	7.72%
When I buy shoes, I check the soles to see if they are slippery.	126	51.22%	57	23.17%	45	18.29%	18	7.32%
I avoid ramps and other slopes.	113	45.93%	61	24.80%	57	23.17%	15	6.10%
I hold onto a handrail when I climb stairs.	141	57.32%	48	19.51%	39	15.85%	18	7.32%
I ask my pharmacist or doctor questions about the side effects of my medications.	40	16.26%	61	24.80%	68	27.64%	77	31.30%
I wear non-slip shoes or non-slip slippers at home.	99	40.24%	71	28.86%	50	20.33%	26	10.57%

**Table 6:** Distribution of responses to social support section.

Question	A Lot		Pretty Often		Sometimes		Rarely		Never	
	N	%	N	%	N	%	N	%	N	%
People show that they care about me.	88	35.77%	84	34.15%	52	21.14%	14	5.69%	8	3.25%
People encouraged me.	76	30.89%	73	29.67%	64	26.02%	19	7.72%	14	5.69%
I got positive comments from people around me.	80	32.52%	79	32.11%	60	24.39%	14	5.69%	13	5.28%
I feel like I belong to a group with similar interests around me.	81	32.93%	66	26.83%	53	21.54%	29	11.79%	17	6.91%
People provided me with helpful information.	87	35.37%	88	35.77%	49	19.92%	17	6.91%	5	2.03%
People give me useful advice when I ask them.	87	35.37%	91	36.99%	42	17.07%	21	8.54%	5	2.03%
If I had a problem, people would share their points of view.	98	39.84%	94	38.21%	35	14.23%	12	4.88%	7	2.85%

**Table 7:** Correlation between fall risk and fall prevention knowledge, behavior, and social support.

		Fall Risk	Knowledge	Behavior	Social Support
Fall risk	r		0.0037	0.3566	0.1180
	*p*		0.9539	<0.0001	0.0647
Knowledge	r	0.0037		0.3640	0.2496
	*p*	0.9539		<0.0001	<0.0001
Behavior	r	0.3566	0.3640		0.3679
	*p*	<0.0001	<0.0001		<0.0001
Social support	r	0.1180	0.2496	0.3679	
	*p*	0.0647	<0.0001	<0.0001	

Note. r: correlation; *p*: probability value.

**Table 8:** A simple linear regression model predicting fall risk based on fall prevention behavior.

Term	Estimate	Std Error	t Ratio	Prob > |t|
Intercept	−0.132	0.930	−0.14	0.8875
Behavior	0.240	0.040	5.96	<0.0001

**Table 9:** Association between age and prevention knowledge, behavior, and social support.

	Age			Monte Carlo *p*-Value Cramer’s V
	65 ≤ 74	75 ≤ 84	≥85	
Knowledge				
Good	50 (70.4%) Exp: 47.61	6 (46.2%) Exp: 8.72	1 (100%) Exp: 0.67	
Fair	15 (21.1%) Exp: 15.87	4 (30.8%) Exp: 2.91	0 (0.0%) Exp: 0.22	Mcp = 0.3495 V = 0.156
Poor	6 (8.5%) Exp: 7.52	3 (23.1%) Exp: 1.38	0 (0.0%) Exp: 0.11	
Behavior				
Positive	31 (15.7%) Exp: 39.90	13 (40.6%) Exp: 6.48	4 (50.0%) Exp: 1.62	
Neutral	158 (80.2%) Exp: 150.45	19 (59.4%) Exp: 24.44	4 (50.0%) Exp: 6.11	Mcp = 0.0087 V = 0.184
Negative	8 (4.1%) Exp: 6.65	0 (0.0%) Exp: 1.08	0 (0.0%) Exp: 0.27	
Social Support				
High	107 (54.3%) Exp: 110.55	22 (68.8%) Exp: 17.96	4 (50.0%) Exp: 4.49	
Fair	89 (45.2%) Exp: 85.62	10 (31.2%) Exp: 13.91	4 (50.0%) Exp: 3.48	Mcp = 0.434 V = 0.074
Poor	1 (0.5) Exp: 0.83	0 (0.0%) Exp: 0.14	0 (0.0%) Exp: 0.03	

Note. Mcp: Monte Carlo *p*-value; V: Cramer’s V.

**Table 10:** Association between gender and prevention knowledge, behavior, and social support.

	Gender				Chi-Square *p*-Value
	Female		Male		
	N	Column %	N	Column %	
Knowledge					$\chi^{2}\;=\;0.3$ *p* = 0.849
Good	64	55.17%	73	56.15%	
Fair	31	26.72%	31	23.85%	
Poor	21	18.10%	26	20.00%	
Behavior					$\chi^{2}\;=\;1.8$ *p* = 0.420
Positive	24	20.69%	24	18.46%	
Neutral	90	77.59%	100	76.92%	
Negative	2	1.72%	6	4.62%	
Social support					$\chi^{2}\;=\;1.4$ *p* = 0.473
High	26	22.41%	38	29.23%	
Fair	52	44.83%	54	41.54%	
Low	38	32.76%	38	29.23%	
Total	116		130		

**Table 11:** Association between educational level and prevention knowledge, behavior, and social support.

	Educational Level								Chi- Square *p*-Value
	No Formal Education		Diploma or Less		Undergraduate Degree		Graduate Degree		
	N	Column %	N	Column %	N	Column %	N	Column %	
Knowledge									$\chi^{2}\;=\;14.0$ *p* = 0.0295
Good	28	38.89%	55	60.44%	37	67.27%	17	60.71%	
Fair	22	30.56%	22	24.18%	11	20.00%	7	25.00%	
Poor	22	30.56%	14	15.38%	7	12.73%	4	14.29%	
				Behavior					$\chi^{2}\;=\;5.6$ *p* = 0.438
Positive	19	26.39%	18	19.78%	8	14.55%	3	10.71%	
Neutral	51	70.83%	71	78.02%	45	81.82%	23	82.14%	
Negative	2	2.78%	2	2.20%	2	3.64%	2	7.14%	
Social support									$\chi^{2}\;=\;5.3$ *p* = 0.499
High	23	31.94%	24	26.37%	12	21.82%	5	17.86%	
Fair	33	45.83%	38	41.76%	22	40.00%	13	46.43%	
Low	16	22.22%	29	31.87%	21	38.18%	10	35.71%	
Total	72		91		55		28		

## Data Availability

The data presented in this study are available in an anonymized form upon reasonable request from the corresponding author owing to ethical restrictions.

## Supplementary Materials

The following supporting information can be downloaded at: https://www.mdpi.com/article/10.3390/healthcare14081109/s1, Table S1: Study instrument, items, responses, and scoring; Table S2: Internal consistency analysis of study scales.
