# Supplementary material for: Assessing Fall Risk, Prevention Knowledge, Behavior and Social Support Among Older Adults: Insights from a Hospital-Based Study in Riyadh
Source: Healthcare (Basel). 2026 Apr 21;14(8):1109. doi: 10.3390/healthcare14081109 (PMC13116690; doi:10.3390/healthcare14081109)
Supplement: Supplementary file 1 [file healthcare-14-01109-s001.zip › healthcare-4186602-supplementary.pdf]

Supplementary Table S1. *Study instrument, items, responses, and scoring.*

No.	Fall Risk Assessment Questions	Response/ Scoring	
		**Yes**	**No**
1	I have fallen at least once in the past year	2	0
2	I use or have been advised to use a cane or walker to get around safely	2	0
3	I feel very steady when I'm walking*	0	1
4	I don't feel the need to hold onto furniture when walking at home*	0	1
5	I am worried about falling	1	0
6	I don't push with my hands to get up from a chair*	0	1
7	I have some trouble stepping up onto a curb	1	0
8	I often have to rush to the toilet	1	0
9	I have lost some feeling in my feet	1	0
10	I take medicine that sometimes makes me feel light-headed or more tired than usual	1	0
11	I take medicine to help me sleep or improve my mood	1	0
12	I often feel sad or depressed	1	0
	**Total out of 14**		

*Reverse-coded items: the response "No" will take 1 point.
The total score is calculated by summing all the "Yes" responses, resulting in a maximum of 14 points, which is then compared to the indicated risk cutoff of 4 points or higher.

No.	Fall Prevention Knowledge Questions	Response/ Scoring		
		**Yes**	**No**	**I don't know**
1	Is it possible to talk to your doctor about your fear of falling or if you feel unsteady?	1	0	0
2	Can the lack of exercise lead to weakness and increase the risk of falling?	2	0	0
3	Do regular eye exams reduce fall risk?	2	0	0
4	Do lighting and handrails on staircases affect fall prevention?	2	0	0
5	Do well-fitted shoes prevent falling?	2	0	0
6	You can prevent falling in the kitchen by keeping things used often on lower shelves.	1	0	0
7	You can prevent falling in the bathroom by putting grab bars next to the bathtub and toilet.	2	0	0
8	You can prevent falling during wadaw' by putting non-slip mats near wadaw' area.	2	0	0
	**Total out of 14**			

Participants scoring of 10 points or higher were classified as having "Good" knowledge. Those scoring between 7 to 9 points were classified as having "Fair" knowledge, and those with of 6 points or fewer were considered to have "Poor" knowledge.

No.	Fall Prevention Behavior Statements	Response/ Scoring			
		All the time	Most days	Some of the days	Never
1	When I stand up, I pause to get my balance	4	3	2	1
2	I talk with someone I know about things I do that might help prevent a fall	4	3	2	1
3	I hurry when I do things*	1	2	3	4
4	I don’t notice if there's a spill on the floor*	1	2	3	4
5	I adjust the lighting at home to suit my eyesight	4	3	2	1
6	When I buy shoes I check the soles to see if they are slippery	4	3	2	1
7	I avoid ramps and other slopes	4	3	2	1
8	I don't worry about walking carefully outside*	1	2	3	4
9	I hold onto a handrail when I climb stairs	4	3	2	1
10	I ask my pharmacist or doctor questions about the side effects of my medications	4	3	2	1
11	I wear non-slip shoes or non-slip slippers at home	4	3	2	1
12	I exercise regularly to improve my balance and strength	4	3	2	1
	**Total out of 36**				

*Reverse-coded items

The minimum and maximum possible scores were 12 and 48. Categories were established by calculating the difference between the minimum and maximum (36 points) and dividing it by 3 for each category: 12-24 points indicated “negative behavior”, 25-36 points indicated “neutral”, and 37-48 points indicated “positive behavior”.

No.	Social Support Statements	Response/ Scoring				
		A lot	Pretty Often	Sometimes	Rarely	Never
1	People show that they care about me	5	4	3	2	1
2	People encouraged me	5	4	3	2	1
3	I got positive comments from people around me	5	4	3	2	1
4	I feel like I belong to a group with similar interests around me	5	4	3	2	1
5	People provided me with helpful information	5	4	3	2	1
6	People give me useful advice when I ask them	5	4	3	2	1
7	If I had a problem, people would share their points of view	5	4	3	2	1

	**Total out of 40**

Each question was rated out of 5, with our cutoff points based on quartiles: (0 - 25%) indicating poor, (26% - 75%) indicating fair, and (76% -100%) indicating high.

Supplementary Table S2. *Internal consistency analysis of study scales*

Items	Item-Total Correlation	Cronbach's Alpha if Item Deleted
**Fall Risk Scale**		
I have fallen at least once in the past year.	0.279	0.551
I use or have been advised to use a cane or walker to get around safely.	0.322	0.541
I feel very steady when I'm walking	0.411	0.520
I don't feel the need to hold onto furniture when walking at home	0.131	0.583
I am worried about falling.	0.345	0.536
I don't push with my hands to get up from a chair	0.187	0.571
I have some trouble stepping up onto a curb.	0.339	0.537
I often have to rush to the toilet.	0.095	0.591
I have lost some feeling in my feet.	0.240	0.560
I take medicine that sometimes makes me feel light-headed or more tired than usual.	0.241	0.560
I take medicine to help me sleep or improve my mood.	0.134	0.579
I often feel sad or depressed.	0.164	0.576
**Knowledge Scale**		
Is it possible to talk to your doctor about your fear of falling or if you feel unsteady?	0.240	0.509
Can the lack of exercise lead to weakness and increase the risk of falling?	0.363	0.459
Do regular eye exams reduce fall risk?	0.325	0.473
Do lighting and handrails on staircases affect fall prevention?	0.205	0.517
Do well-fitted shoes prevent falling?	-0.019	0.575
You can prevent falling in the kitchen by keeping things used often on lower shelves.	0.286	0.489
You can prevent falling in the bathroom by putting grab bars next to the bathtub and toilet.	0.314	0.483
You can prevent falling during wadaw' by putting non-slip mats near wadaw' area.	0.295	0.490
**Behavior Scale**		
When I stand up, I pause to get my balance.	0.314	0.383
I talk with someone I know about things I do that might help prevent a fall.	0.181	0.426
I hurry when I do things.	0.002	0.480
I don't notice if there's a spill on the floor	-0.117	0.518
I adjust the lighting at home to suit my eyesight.	0.149	0.436
When I buy shoes I check the soles to see if they are slippery.	0.352	0.376
I avoid ramps and other slopes.	0.356	0.375
I don't worry about walking carefully outside	-0.006	0.484
I hold onto a handrail when I climb stairs.	0.346	0.377
I ask my pharmacist or doctor questions about the side effects of my medications.	0.098	0.452
I wear non-slip shoes or non-slip slippers at home.	0.401	0.357
I exercise regularly to improve my balance and strength.	0.063	0.461
**Social Support Scale**		
People show that they care about me.	0.656	0.866
People encouraged me.	0.743	0.854

I got positive comments from people around me.	0.677	0.863
I feel like I belong to a group with similar interests around me.	0.514	0.887
People provided me with helpful information.	0.720	0.858
People give me useful advice when I ask them.	0.717	0.858
If I had a problem, people would share their points of view.	0.686	0.862
